# Supplementary material for: The impact of temporal sampling resolution on parameter inference for biological transport models
Source: PLoS Comput Biol. 2018 Jun 25;14(6):e1006235. doi: 10.1371/journal.pcbi.1006235 (PMC6034909; doi:10.1371/journal.pcbi.1006235)
Supplement: S1 Appendix — Description of simulations to verify the analytic form for the emission probabilities. (PDF) [file pcbi.1006235.s001.pdf]

The impact of temporal sampling resolution on parameter inference for biological transport models: Supplementary material

Jonathan U. Harrison* [1], Ruth E. Baker[1],

[1] Wolfson Centre for Mathematical Biology, Mathematical Institute, University of Oxford, United Kingdom

* harrison@maths.ox.ac.uk

S1 Appendix

Comparison with simulations

To verify our emission probabilities calculated in the Section ‘Derivation of emission probabilities’, we can compare the distributions of observed angle changes from simulations with those predicted theoretically. We simulate from the VJP model for a known pattern of hidden states and assume no measurement noise is present. Performing these checks for hidden states of the form $0, 1$, we obtain results as shown in Fig 2Sa); there is excellent agreement between simulation and theory which verifies our theoretical results. For hidden states of the form $1, 0$, we must condition on the value of the previous angle change. We demonstrate agreement between the theory and simulations, for different values of the previous angle change, in Fig S2b). Similarly, we show corresponding results for hidden states of the form $1, 1$ in Fig S2c).
