# Supplementary figures and images for: The impact of temporal sampling resolution on parameter inference for biological transport models
Source: PLoS Comput Biol. 2018 Jun 25;14(6):e1006235. doi: 10.1371/journal.pcbi.1006235 (PMC6034909; doi:10.1371/journal.pcbi.1006235)

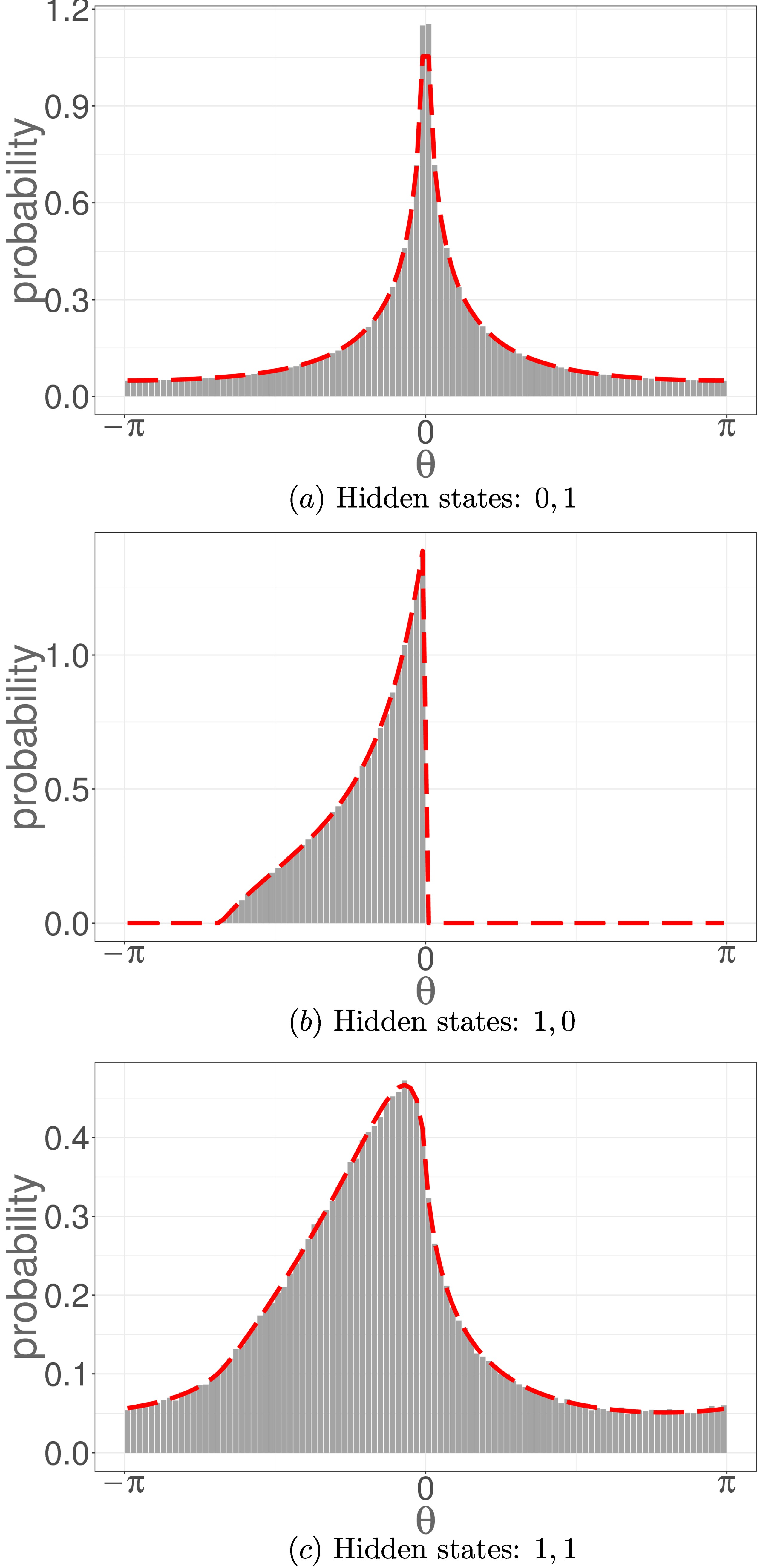

Supplement: S1 Fig — Comparison between simulated results and theoretical predictions of the observed angle change for hidden states of the form 0, 1 in a), 1, 0 in b), and 1, 1 in c). The simulated results are shown by the histogram and the theoretical prediction for the observed angle change distribution is shown as the red dashed line. For both b) and c), we have conditioned on an observed angle change in the previous time interval of 0.1 rad, and for c) we also conditioned on an observed angle change prior to that of −1.0 rad. To generate these results, we used N = 107 simulated trajectories with running speed c = 50μms−1, uniform reorientation kernel, reorientation rate λ = 0.2 s−1 and time discretization Δt = 1 s. (TIF) [file pcbi.1006235.s002.tif]

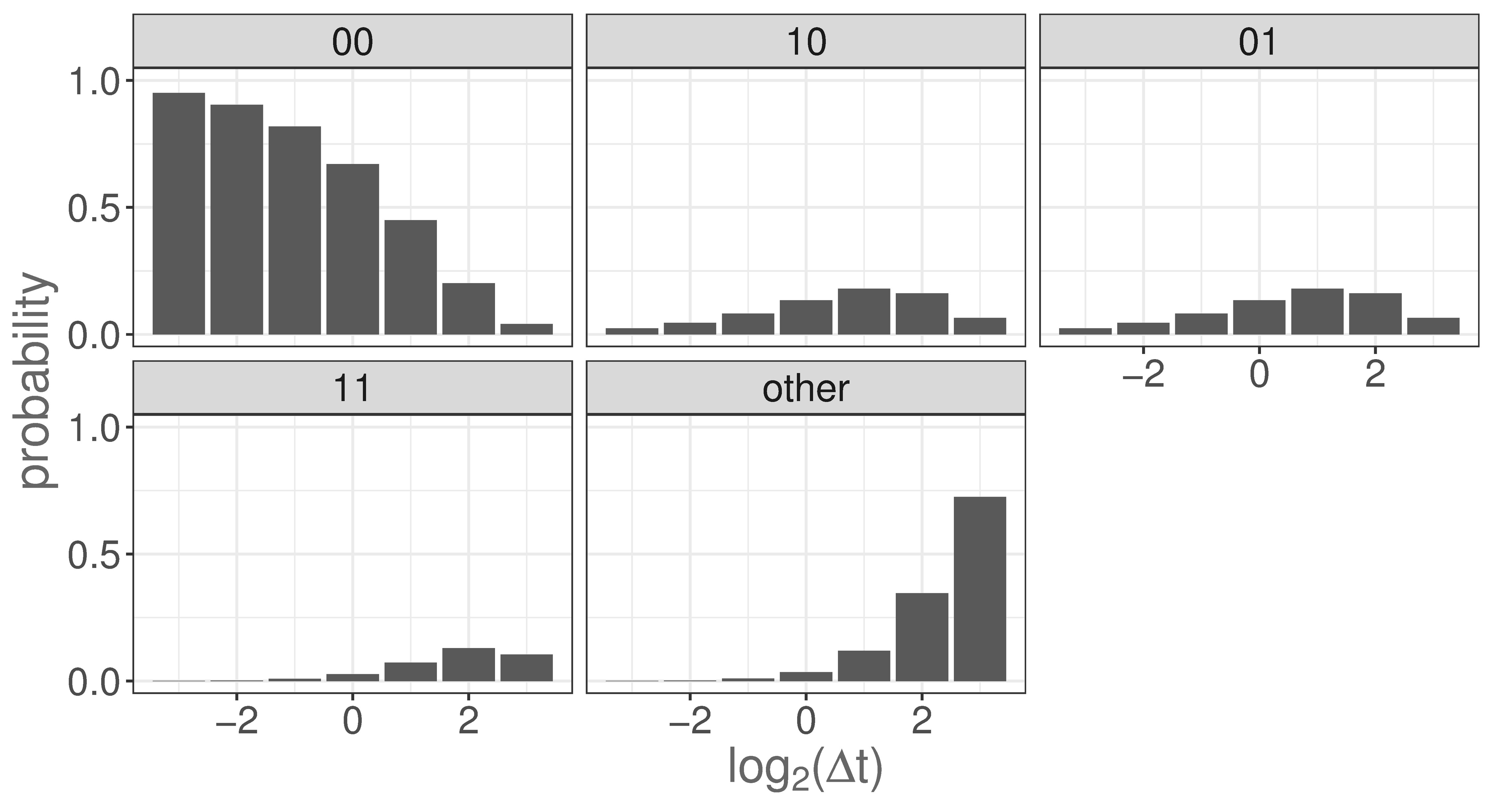

Supplement: S2 Fig — The probability of sequences of hidden states as Δt varies with reorientation rate λ = 0.2 s−1. For large values of Δt, the assumptions of the model start to break down as multiple reorientations appear within a single time interval. (TIF) [file pcbi.1006235.s003.tif]

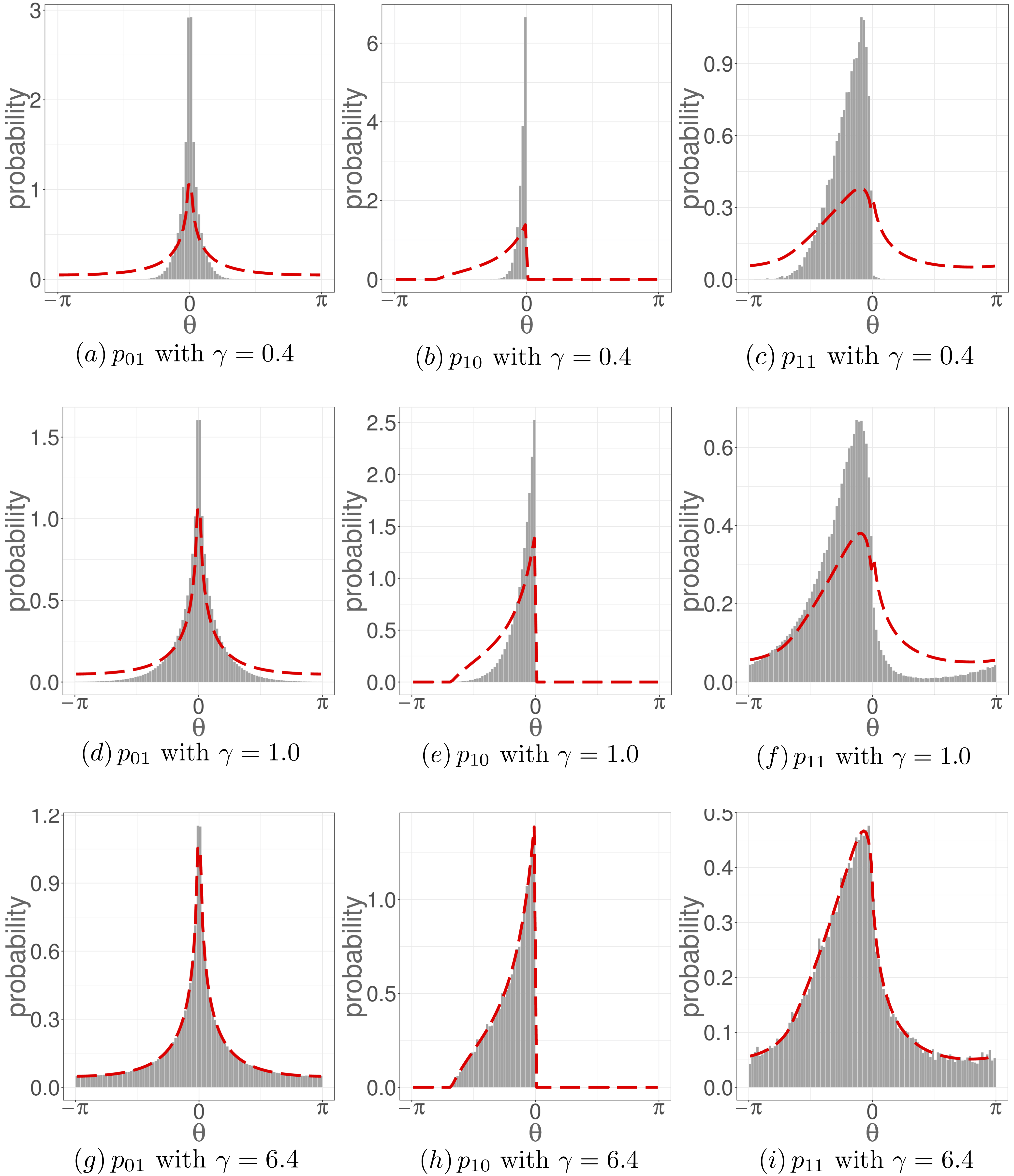

Supplement: S3 Fig — Comparison between assumed distribution of observed angle changes and simulated distributions when using a misspecified model for the reorientation kernel. We simulate N = 107 angle changes using a wrapped normal reorientation kernel with dispersion parameter γ given a certain sequence of hidden states (0, 1 in a), d), g); 1, 0 in b), e), h); 1, 1 in c), f), i)) and show a grey histogram of the simulated observed angle changes. To demonstrate the misspecification in the emission probabilities, we plot the assumed theoretical distribution of the observed angle changes as a red dashed line, based on assuming that the reorientation kernel is a uniform distribution. The model is more misspecified for a smaller value of the dispersion parameter γ. As in S1 Fig, we have conditioned on an observed angle change in the previous time interval of 0.1 rad for b), c), e), f), h), and i). For c), f), and i), we also conditioned on an observed angle change prior to that of −1.0 rad. We used a dispersion parameter, γ, in the reorientation kernel of γ = 0.4 for a), b), and c), γ = 1 for d), e), and f), and γ = 6.4 for g), h), and i). (TIF) [file pcbi.1006235.s004.tif]

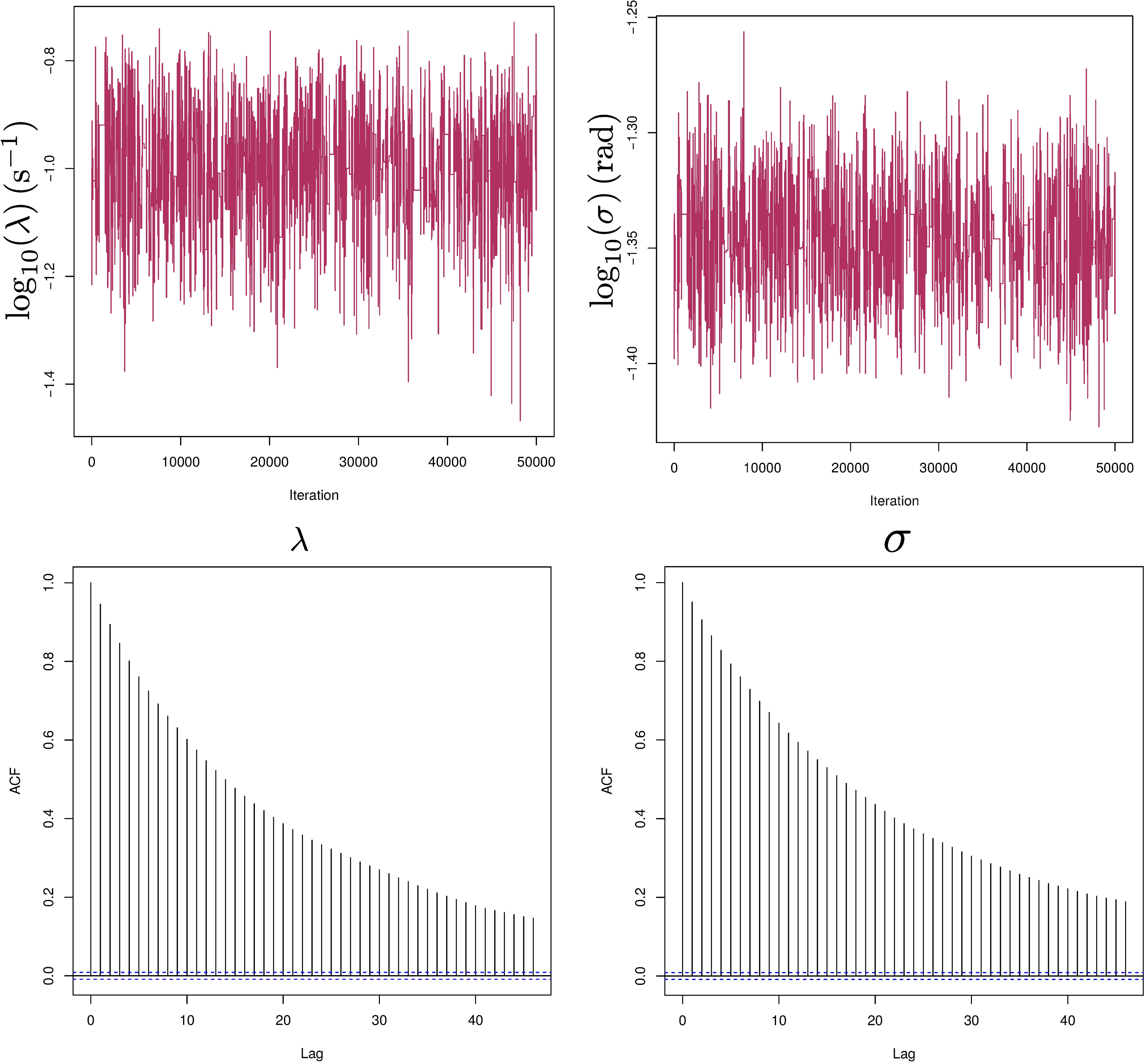

Supplement: S4 Fig — Traceplots and autocorrelation functions for MCMC chains to analyse convergence are shown for data generated with parameters λ = 0.2 s−1, Δt = 0.25 s, σ = 0.04 rad, as for the first posterior shown in Fig 7a). Similar results are seen in sampling for other posterior distributions shown. (TIF) [file pcbi.1006235.s005.tif]

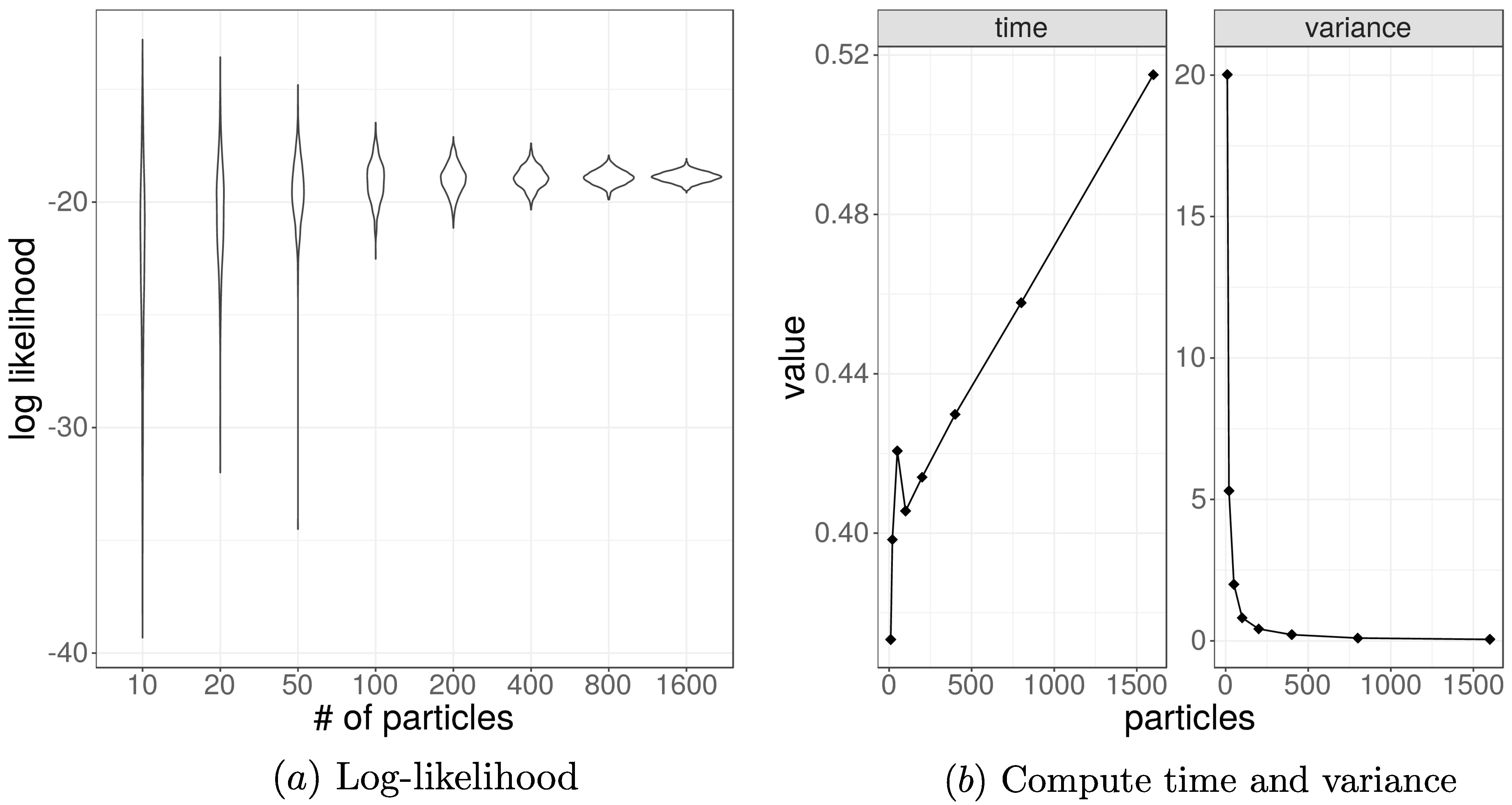

Supplement: S5 Fig — The number of particles used in the particle filter affects the variance of estimates of the log-likelihood. However, a higher computational cost is required to use more particles. In a), we show how the distribution of the log-likelihood estimates varies (provided the filter does not become degenerate) as we change the number of particles. We use 1000 runs of the particle filter with the specified number of particles and estimate the log-likelihood at the true value of the parameters (λ = 0.2 s and σ = 0.08 rad) used to generate a synthetic dataset. In b), we illustrate the variance in the (nondegenerate) log-likelihood estimates, and the mean time to obtain a single estimate. A moderate increase in the compute time to run the particle filter offers substantial decrease in the variance of the log-likelihood estimates. To strike a reasonable balance, we use 400 particles to generate the results presented in this work. (TIF) [file pcbi.1006235.s006.tif]

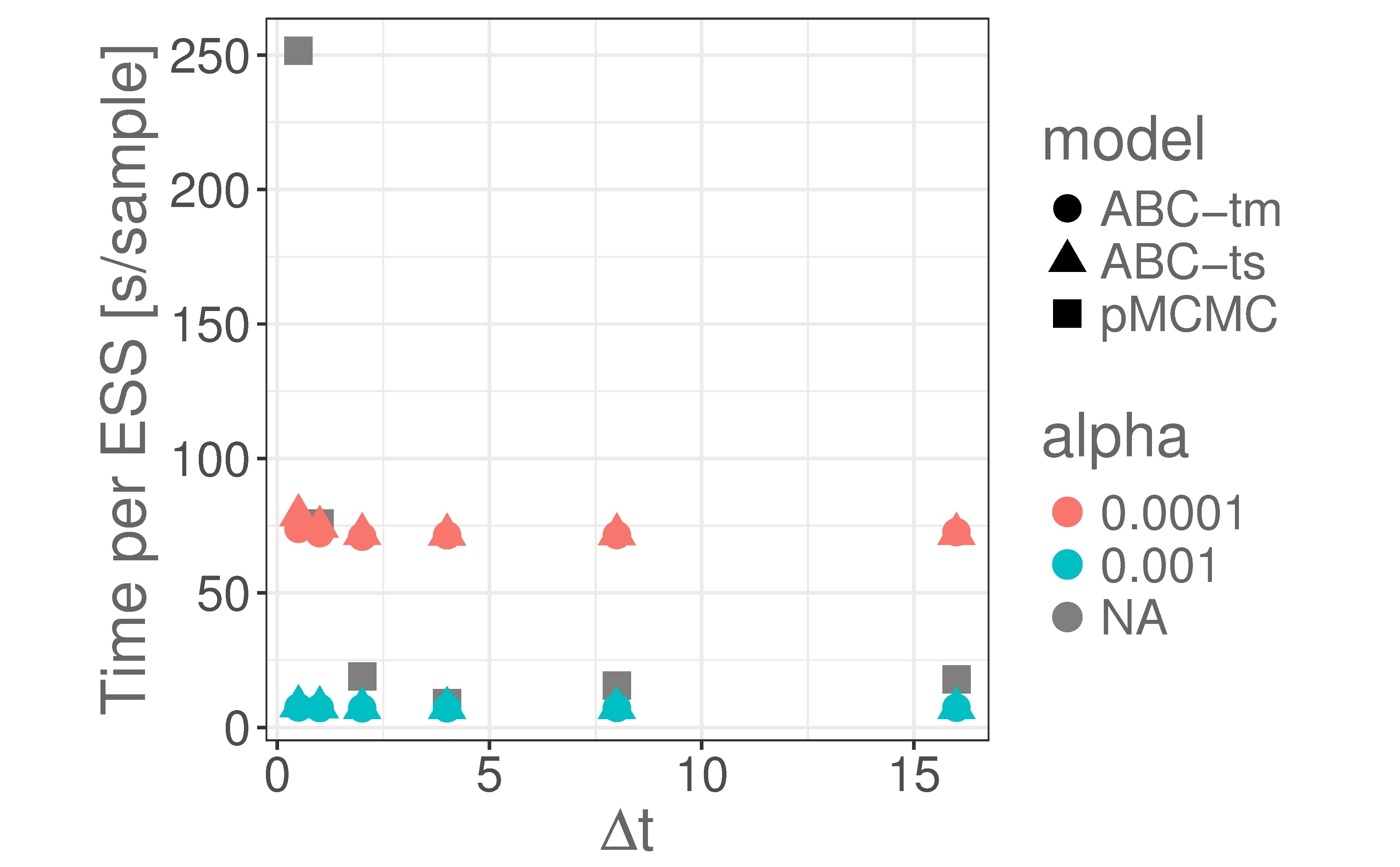

Supplement: S6 Fig — The computational cost of parameter estimation with pMCMC and ABC per effective sample size depends on Δt. We quantify here a comparison between the computational cost of the pMCMC and ABC methods for parameter estimation when varying Δt. Datasets were generated with σ = 0.04 rad as in Fig 7a). The cost to produce a sample via pMCMC increases as Δt decreases, as shown by the grey squares. The cost for ABC remains approximately constant with Δt as we simulate data from the model a fixed number of times, N. The acceptance rate, α, in ABC affects the sample size we produce for a fixed number of simulations, N. For the results in Fig 10, an acceptance rate of α = 0.1% was used. This gives a cost per sample lower than for pMCMC, shown by the blue circles and triangles. For a smaller acceptance rate, α = 0.01% (shown by red circles and triangles), the cost per sample is much higher and the plots of the posterior are unchanged compared to those for α = 0.1%. The results for pMCMC are given by squares, ABC with transition matrix summary statistics are shown as circles and ABC with time series summary statistics are shown as triangles. We note that the computational cost depends strongly on the problem considered and the implementation used. (TIF) [file pcbi.1006235.s007.tif]
